# Why Do We Need Media Multitasking? A Self-Regulatory Perspective

**DOI:** 10.3389/fpsyg.2021.624649

**Published:** 2021-02-11

**Authors:** Agnieszka Popławska, Ewa Szumowska, Jakub Kuś

**Affiliations:** ^1^Faculty of Psychology in Sopot, SWPS University of Social Sciences and Humanities, Sopot, Poland; ^2^Institute of Psychology, Department of Philosophy, Jagiellonian University, Kraków, Poland; ^3^Faculty of Psychology in Wrocław, SWPS University of Social Sciences and Humanities, Wrocław, Poland

**Keywords:** media multitasking, multitasking performance, self-regulation, performance strategy, multitasking effectiveness

## Abstract

In the digital world of today, multitasking with media is inevitable. Research shows, for instance, that American youths spend on average 7.5 h every day with media, and 29% of that time is spent processing different forms of media simultaneously (Uncapher et al., 2017). Despite numerous studies, however, there is no consensus on whether media multitasking is effective or not. In the current paper, we review existing literature and propose that in order to ascertain whether media multitasking is effective, it is important to determine (1) which goal/s are used as a reference point (e.g., acquiring new knowledge, obtaining the highest number of points in a task, being active on social media); (2) whether a person's intentions and subjective feelings or objective performance are considered (e.g., simultaneous media use might feel productive, yet objective performance might deteriorate); and finally (3) whether the short- or long-term consequences of media multitasking are considered (e.g., media multitasking might help attain one's present goals yet be conducive to a cognitive strategy that leads to lesser attentional shielding of goals). Depending on these differentiations, media multitasking can be seen as both a strategic behavior undertaken to accomplish one's goals and as a self-regulatory failure. The article integrates various findings from the areas of cognitive psychology, psychology of motivation, and human-computer interaction.

## Introduction

In the digital world of today, multitasking with media is inevitable. For instance, research shows that American youths spend on average 7.5 h every day with media, and 29% of that time is spent processing different forms of media simultaneously (Uncapher et al., 2017). Another study showed that American adults often engage in two additional media-related activities when reading, watching TV, or listening to news (Ran et al., 2016). According to a survey by Pew Research Center, 95% of teens have access to a smartphone, and 45% report they are online “almost constantly” (Anderson and Jiang, 2018). Moreover, research shows that preschoolers are exposed to digital devices before they are introduced to books (Hopkins et al., 2013), and bundle multitasking among college students (i.e., multitasking with more than three activities at the same time) is almost as frequent as performing two activities simultaneously (David et al., 2014).

This increase in media multitasking has spurred scientific interest in this phenomenon. Indeed, recent years have seen more and more studies focusing on the effect media multitasking has on people's performance (e.g., Ophir et al., 2009; Alzahabi and Becker, 2013; Minear et al., 2013; Sanbonmatsu et al., 2013). Despite numerous investigations, however, there is no consensus on whether media multitasking is effective or not. Some studies show that it is related to poorer performance (e.g., Ophir et al., 2009; Oviedo et al., 2015; Kazakova et al., 2016; Bellman et al., 2017); however, according to other reports the opposite seems to be the case (e.g., Adler and Benbunan-Fich, 2012; Lui and Wong, 2012), and yet others show no significant relationship at all (e.g., Law and Stock, 2017).

This topic seems to be even more complex as media multitasking is associated with several paradoxes. Research shows, for instance, that those who engage in media multitasking might be the ones who are bad at it—the “multitasking paradox” (e.g., Ophir et al., 2009). People increasingly multitask, especially with digital technologies, despite the fact that doing so causes their performance to deteriorate—the so called “myth of multitasking” (Rosen, 2008; Wang and Tchernev, 2012).

We propose that in order to understand these paradoxes, it is important to take a multitasker's goals into account. We thus adopt a self-regulatory perspective which assumes that people's behavior is goal-directed and they take actions in order to attain desired outcomes and/or avoid undesired outcomes (e.g., Austin and Vancouver, 1996; Carver and Scheier, 1998; Kruglanski et al., 2002, 2015; Neal et al., 2017; Kruglanski and Szumowska, 2020). In other words, people's behavior and the tasks and activities they perform serve as means to achieve their goals and needs (Kruglanski et al., 2002, 2015; Ajzen and Kruglanski, 2019; Szumowska and Kruglanski, under review). Self-regulation is the dynamic process by which people manage these tasks and activities as they strive to achieve desired outcomes or avoid undesired outcomes, and as such it is crucial for successful goal attainment (e.g., Austin and Vancouver, 1996; Fishbach et al., 2006; Neal et al., 2017).

In what follows, we review recent findings and the existing literature on multitasking with media; we propose that in order to ascertain whether media multitasking is effective or not, it is necessary to answer the important questions below.

1. What goals do we take into account when assessing the effectiveness of media multitasking? We can imagine a situation in which someone tries to learn something new and at the same time is texting with friends. From one point of view, the learning process could be impaired but the need for social contact is fulfilled. Should we say in this case that media multitasking is not efficient? It just depends on the interpretation and the evaluation criterion.
2. How is performance defined and measured? Do we take into account objective or subjective measures? We can focus on task performance indices such as accuracy, speed, recall and memory, and the number of errors; on the other hand, we can consider the “feeling of productivity” which a person who multitasks can obtain. These different indices, when used, can lead to different conclusions.
3. Are short- or long-term consequences considered? Today's generation, which has been brought up with smartphones in their hands, experiences the multitasking mode in a completely different way. In the short-term context, media multitasking may be treated as a way to fulfill certain needs, but in the long-term context it is not known how this mode will affect cognitive and emotional functioning. Some studies suggest that it may be detrimental to cognitive function (Loh and Kanai, 2014; van der Schuur et al., 2015). Others argue that although media multitasking can be detrimental to immediate performance, it can enhance learning in the long run (Hwang et al., 2014; Wang et al., 2015).

This article thus integrates various findings from the areas of cognitive psychology, psychology of motivation, media research and human-computer interaction. It also provides an integrative view on a very broad and extensive body of literature and proposes criteria which, when applied, help explain (at least some) inconsistencies found in the media multitasking literature. This seems especially relevant given that the field of studies on media and digital multitasking is growing very fast and there is a need to systematize the often mixed results. We propose three systematizing criteria we find especially useful in understanding the research findings on media multitasking and by doing so we encourage further attempts.

We discuss the three criteria in more detail in the parts that follow. First, however, we define multitasking and review the recent findings on its effects.

## Media Multitasking and Its Effects

Multitasking is defined as undertaking various activities in the same time period, each of which has its own purpose and requires attention (Adler and Benbunan-Fich, 2012; Szumowska and Kruglanski, under review). As such, it occurs when any two or more activities are combined. In this article, we focus on a specific kind of multitasking, namely media multitasking [e.g., Bardhi et al. (2010), Uncapher et al. (2017), and Szumowska et al. (2018)]. A person who functions in a situation of heavy media multitasking uses multiple media channels simultaneously. This might involve various devices, such as using the internet on a laptop and listening to the radio, or a single device, such as a computer screen with multiple browser tabs open, which is now a very common practice. So, in this article, media multitasking is understood as electronic device-based activity that can involve electronic devices only or electronic devices combined with some offline activities. As an illustrative example, just imagine students listening to a lecture whilst constantly checking notifications on their phones.

A related term, polychronicity, can also be found in the literature [e.g., König and Waller (2010), Poposki and Oswald (2010), and Capdeferro et al. (2014)]. It is defined as a natural tendency to structure time in a multitasking manner, or a preference to perform several activities simultaneously (König and Waller, 2010; Capdeferro et al., 2014). Initially, the term described the degree to which people or cultures do several things at the same time [as opposed to doing one thing at a time (Hall, 1959)]. However, recently researchers postulated that the term *polychronicity* should only be used to describe the preference for doing several things at the same time, whereas the behavioral aspect of polychronicity should be referred to as multitasking (König and Waller, 2010). Therefore, we focus on multitasking, particularly media multitasking.

The media multitasking phenomenon is all the more noteworthy because it has become a predominant media-use behavior, particularly among adolescents (Brown and Cantor, 2000; Rideout et al., 2010; Jacobsen and Forste, 2011; Wood et al., 2012; Voorveld and van der Goot, 2013). As the results of Judd and Kennedy (2011) show, the vast majority of students engage in both task-switching and multitasking behaviors.

Due to the great popularity of media multitasking, numerous studies have looked into its relations with performance and have tried to determine whether frequent media multitaskers are more or less effective in cognitive functioning. In this kind of research, classic cognitive tasks are typically used (e.g., N-back task, dual task, cognitive control task etc.). Researchers test whether participants can effectively use their cognitive resources under media multitasking of varying frequency (e.g., Ophir et al., 2009; Wallis, 2010; Cain and Mitroff, 2011; Baumgartner et al., 2014; Uncapher et al., 2017). So far there is no clarity as to whether people who frequently engage in media multitasking are less effective than people who engage in media multitasking less frequently. A classic experiment conducted by Ophir et al. (2009) showed that participants with a high level of media multitasking (heavy media multitaskers) are less effective in various distractor-filtering tests than people with low levels of media multitasking (light media multitaskers). Similar results were obtained by Sanbonmatsu et al. (2013), who showed that heavy media multitaskers are more vulnerable to external distractors. However, not all studies have shown an association between media multitasking and distractibility. The replication studies and meta-analysis conducted by Wiradhany and Nieuwenstein (2017) showed that there is no significant association between media multitasking and distractibility. The authors asked whether this kind of association can be observed in laboratory-based information-processing tasks. Thus, our review of research focused on measuring cognitive function does not give a clear answer as to whether media multitasking is an effective way to function or not, especially when we take functioning in a real-life environment into account.

Another body of research that attempts to determine whether multitasking impairs cognitive functioning is related to learning processes. This research tests whether media multitasking hinders acquisition of new knowledge. A literature review conducted by May and Elder (2018) indicates that media multitasking during lectures impairs the learning process due to the lack of attention that is paid to the presented information. Such a negative effect was observed in test performance, grades, recall and note taking. It is worth emphasizing that the media multitasking effect was often observed after a short time span. In a real-life study, Srivastava (2013) showed that introducing time limits caused significant performance impairments in a media multitasking condition compared to the control group. The same pattern of results was observed in experiments in which participants were asked to pay attention to a lecture and simultaneously answer text messages or use a laptop or (in the control group) to only focus on the lecture (Sana et al., 2013; Dietz and Henrich, 2014; Gupta and Irwin, 2016). The main conclusion of these experiments is that media use impairs the learning process, during both learning and studying (Law and Stock, 2017). This effect concerns not only students using a laptop during a simulated lecture but also those sitting nearby (Sana et al., 2013).

In contrast, Aagaard (2019) described experiments in which participants in the control group read a text, whereas participants in the experimental group read the same text and were engaged in instant messaging at the same time. An interesting finding was that both groups were equally effective in performance of the task, but the media multitasking group needed more time to accomplish it. In other words, additional time was needed to compensate for the instant messaging interruptions. This suggests that, if given additional time, heavy media multitaskers could be just as efficient as light media multitaskers (see also Fox et al., 2009). In another study, Law and Stock (2017) found that students who demonstrate a deep approach to learning also engage more deeply in learning technologies: they reported spending more time reading printed media and using email and “other” computer applications such as Microsoft Word. The results lead to the conclusion that it might be better to promote active learning in the classroom by using modern devices rather than trying to forbid their use.

More specific areas of research related to individual differences, such as working memory [e.g., Ophir et al. (2009), Colom et al. (2010), and Sanbonmatsu et al. (2013)], also play a significant role in determining the effects of media multitasking on cognitive functions. Many of the results of these studies show a kind of “multitasking paradox”: those who are most capable of multitasking effectively are not the ones who are most likely to engage in multiple tasks simultaneously (Ophir et al., 2009; Sanbonmatsu et al., 2013). It is particularly interesting that Sanbonmatsu et al. (2013) showed that there is a positive correlation between participants' own belief in their multitasking ability and their media multitasking activity (frequency of engagement in simultaneous media use). Media multitasking activity, however, was negatively associated with actual media multitasking ability [as measured with the Operation Span task (Engle, 2002)]. This means that a person may not have a cognitive predisposition for such functioning even though they feel that it is a style of working that serves them.

What follows from the brief overview of the recent studies presented above is that, regardless of the analyzed area, there is no consensus as to whether media multitasking impairs cognitive functioning or not. So, we propose that instead of trying to provide an unequivocal answer to this question, it might be more useful to ask when or under what conditions media multitasking is effective and which reference point or goal we use to judge its effectiveness. Particularly, we need to be mindful of the fact that people have multiple goals at the same time [e.g., Kruglanski et al. (2002, 2015)]; therefore, we need to be specific about the goal(s) we refer to when assessing the efficiency of multitasking. Moreover, we need to be clear about how efficiency (performance) is measured and which consequences (short or long term) we are interested in. We discuss these aspects in the sections to follow.

## What Goal(s) Are Used as a Reference Point?

As we mention above, there is no clarity as to whether media multitasking is an effective way of functioning or not. However, as we argue here, it might depend on which goal or goals are used as a reference point. Multitasking, by definition, involves more than one task, each of which is associated with different (sub)goals (Adler and Benbunan-Fich, 2012; Szumowska and Kruglanski, under review). Media multitasking and multitasking in general can have different effects on the performance of these different tasks. It can disrupt performance on one task (e.g., a more demanding activity which requires more resources). At the same time, it may not affect the other task(s) or may affect them positively. So, our conclusion concerning the effects of multitasking will depend on which tasks and goals are treated as a reference point.

This might be more evident when one goal is more important than another and one task can be easily identified as the primary one and the other as a secondary one, in which case the more important goal will probably serve as a reference point. This is often the case in learning scenarios, when one goal is an academic goal related to acquiring knowledge, and the other goal is related to engagement in media-related activities (e.g., texting, checking social media) (Dietz and Henrich, 2014; Gupta and Irwin, 2016). Since the learning goal is more important (at least in the academic context), and engagement in additional activities often impairs it, the conclusion that often follows is that media multitasking leads to poorer performance. However, when one does not care about the given learning goal and has important activities to attend to online, should the effectiveness of multitasking be judged only on the basis of one's performance of the learning task? We think not, and taking a one-goal perspective could be responsible for the differences in conclusions regarding the effectiveness of media multitasking, especially regarding the discrepancy between laboratory studies and people's everyday experience. For example, even though an individual's in-class learning might have been impaired due to involvement with media, during the class they dealt with several other tasks (e.g., communicated with family or friends, shopped online, or looked at the news or information for another class). So, a person's *overall* performance (performance on all active goals the person has) might be better when multitasking, not poorer.

On the other hand, the importance of the current goals is a factor that determines whether people engage in multitasking in the first place, and to what extent they do so. In a series of studies, Szumowska and Kruglanski (under review) showed that the more important a given goal is to a person, the less likely they are to engage in multitasking. For instance, in one study (Study 6) the researchers showed that the more important performing well in a given class was to students, the less likely they were to engage in media multitasking with smartphones during that class. A similar relationship was proposed by Judd and Kennedy (2011), who suggested that students with specific goals (i.e., studying for a difficult exam) may be less eager to multitask than students with less consequential goals (i.e., communication with friends for fun).

However, the more active goals of equal importance people have, the more likely they are to multitask (Szumowska and Kruglanski, under review). This shows that people multitask to attain multiple goals and meet the requirements of multiple tasks. This multiplicity should not be omitted when analyzing performance efficiency. Therefore, rather than focusing only on one task, analyzing aggregated performance on all active tasks might be a more adequate solution.

This is important given that much of the previous research on multitasking focused on the costs (i.e., inefficiencies) that arise when two tasks need to be performed in a given period, as compared to a situation in which only one of the two tasks is performed in the same period. A multitude of studies show that performance costs occur under multitasking (vs. monotasking). For instance, response times increase when stimuli for two tasks are presented concurrently, compared to a situation in which only one stimulus is presented [dual-tasking costs (Pashler, 1994)], or when one switches between tasks compared to a situation in which one repeats the same task over and over again [task-switching costs (Rogers and Monsell, 1995; Monsell, 2003)]. This suggests that multitasking is inefficient and people should eschew engaging in it.

However, this depends on a reference point. In a typical dual-task scenario, response times under dual tasking are compared to response times under monotasking, i.e., only one task (goal) is treated as a reference point. However, when we analyze the same situation from the perspective of two active goals, multitasking might be more efficient than monotasking. This is because when a task is combined with another task, performing it might take more time than performing one task at a time but less than when performing the two tasks consecutively (the time needed to complete the combined tasks, even when burdened with dual-tasking costs, might be less than the sum of the times needed to perform the two tasks consecutively).

To sum up, media multitasking can be either productive or not, depending on the goals one takes into account and their importance [see also Szumowska and Kruglanski (2019)]. In line with that, Duff and Sar (2015) propose that recall and recognition will be higher if the respondent is more highly motivated to multitask.

## How is Performance Defined and Measured?

### Are Subjective or Objective Performance Criteria Analyzed?

Another important issue when determining whether media multitasking is effective or not is how performance is defined and measured. An important differentiation is whether a person's intentions and subjective feelings or objective performance are considered: simultaneous media use might *feel* productive, yet objective performance might still be worse. As discussed in the previous section, multiple studies show that high levels of media multitasking are associated with worse performance, as measured with task performance indices such as accuracy, speed, number of errors etc. For instance, media multitasking has been associated with lower performance on task-switching and working memory tasks (Ophir et al., 2009), and poorer processing of advertisements and their impaired message recall and recognition. Worse performance was also observed in such simultaneous activities as watching TV episodes and engaging in Facebook activities (Oviedo et al., 2015), or reading online articles and listening to a podcast (Srivastava, 2013), social TV viewing (Bellman et al., 2017), watching TV advertisements and solving anagrams (Segijn et al., 2017), watching TV advertisements and website advertisements (Kazakova et al., 2016) [see Garaus (2019), for an overview]. Similar results have been found in classic studies on multitasking, where the usual finding is that performing several activities at the same time (or frequently switching between them) leads to more errors, distraction, interference, and lost time as compared to a situation in which these activities are performed one at a time [see Pashler (1994), Monsell (2003), Courage et al. (2015), for overviews]. Some researchers even argue that multitasking, and digital multitasking especially, seems to (temporarily) reduce people's cognitive capacity and make “smart people underperform” (Hallowell, 2005).

This contradicts why people engage in multitasking in the first place. In line with the definition, they do so in order to attain several goals within one time period [e.g., Adler and Benbunan-Fich (2012) and Szumowska and Kruglanski (under review)], i.e., they do so to be more productive and efficient. Indeed, researchers have argued that switching between tasks contributes to the “illusion of productivity” (Turkle, 2012; Adler and Benbunan-Fich, 2013); that is, people *feel* productive while multitasking. Wober (1992) showed, for instance, that children believe that background television helps them work efficiently. The children in Patton et al. (1983) study believed in the beneficial influence of radio programs while doing math homework. According to Lin et al. (2015), teenagers and young adults are convinced that they are productive while media multitasking. In Clayson and Haley (2012) study, the majority of students claim that they are able to pay attention to a lecture and send text messages at the same time (but those who were in this condition received lower grades). Also, Sanbonmatsu et al. (2013) found that people's perception of their multitasking ability is significantly inflated. These studies show that people think they are efficient while multitasking—more efficient in fact than when they perform tasks separately.

Interestingly, Srna et al. (2018) additionally showed that construing a given activity as multitasking increases not only the perception of productivity but also productivity itself. These authors argue that the same activity can often be seen as single-tasking or multitasking (e.g., note taking during a lecture can be seen as one activity or two separate activities: one related to listening and one to taking notes). This matters for performance. Across 32 studies, these researchers found that individuals who perceived a given activity as multitasking were more engaged and consequently outperformed those who perceived that same activity as single-tasking.

### What Performance Metrics Are Used?

In reference to objective as well as subjective performance, different metrics can be used and media multitasking can be differently related to each of them. In one study, media multitasking was positively related to creativity but negatively to accuracy (Adler and Benbunan-Fich, 2012). Specifically, participants who could freely switch between tasks demonstrated higher creativity in imagining solutions but performed more poorly when performance was indexed with accuracy. Voorveld (2011) conducted an experiment in which participants processed an internet website and a radio commercial. The results showed that affective and behavioral responses were better in the condition in which respondents were exposed to both media rather than only one medium, but media multitasking had a negative effect on the recollection and recognition of auditory information. Smit et al. (2017) asked participants to perform an internet search task while listening to related radio commercials. Compared to unrelated radio commercials, they reported a positive influence on brand evaluation, recall and recognition. Studies by May and Elder (2018) showed a reduction in efficiency due to media multitasking, but there were no effects on comprehension. After an interruption caused by media multitasking, participants could return to and read some parts of the material again. Thus, comprehension was not affected. Yet, this process needs more time, which often is limited, therefore participants who media multitask may be less efficient.

The above results demonstrate that effectiveness can be defined using different criteria. Depending on these criteria, different conclusions might be drawn as to whether media multitasking is effective or not.

## Are Short- or Long-Term Consequences Considered?

The studies published so far show that the impact of media multitasking on cognitive functioning is ambiguous. Some results indicate, for example, a short-term increase in creative thinking [e.g., Adler and Benbunan-Fich (2012)], but on the other hand they show that multitasking can lead to problems with concentration [e.g., Judd and Kennedy (2011)]. However, all these results are rather situational and quite short; thus, it seems reasonable to also ask about the long-term effects of multitasking. The meta-analyses published so far indicate that this impact is rather negative and concerns mainly such areas as cognitive control and academic performance (van der Schuur et al., 2015). Ophir et al. (2009) postulated that frequent engagement in media multitasking might create a different cognitive orientation or cause people to process information differently. The researchers argued that the breadth-biased media consumption behavior of heavy media multitaskers is mirrored by their breadth-biased cognitive control, as reflected in greater susceptibility to interference from irrelevant environmental stimuli and from irrelevant representations in memory [however, Minear et al. (2013), found no similar effects, and Szumowska et al. (2018) identified the moderators of the relationship].

Newport (2016) additionally indicates that too-frequent multitasking may lead to the inability to think deeply and critically in the long run. Moreover, Loh and Kanai (2014) demonstrated that more intense media multitasking activity is associated with smaller gray-matter density in the anterior cingulate cortex (correlational evidence). So, if multitasking relates to changes at the neural level, it is all the more appropriate to formulate questions about whether and how deeply it can transform cognitive functioning.

Such questions seem even more worthwhile given that positive long-term effects of media multitasking have been postulated as well. For instance, researchers have argued that although media multitasking can be detrimental to immediate performance, it can enhance learning in the long run [Hwang et al., 2014; Wang et al., 2015; see also Szumowska et al. (in press), for no effects of media multitasking].

It thus seems that it will take several more years to see how multitasking has actually affected the cognitive systems of the current generation, which multitasks on a daily basis (Näsi and Koivusilta, 2013). It seems, therefore, that while the short-term consequences of multitasking may be significant in some situations, many more studies, including longitudinal ones, are still needed to see what impact multitasking with media will have in a broader sense.

## Media Multitasking as Strategic Behavior

It can be also considered that media multitasking, and multitasking in general, is strategic in the sense that it serves the important goals of an individual, albeit not always conscious ones [e.g., Wang and Tchernev (2012) and Szumowska and Kruglanski (under review)]. In line with this assumption, people multitask to optimize resource allocation and maximize their progress on multiple goals (e.g., González and Mark, 2005).

This is clearly seen in the domain of educational or occupational goals. Researchers argue that the majority of computer use is in fact media multitasking (Carrier et al., 2009). A lot of this use is in the professional settings in which people perform their job duties, and in order to do so efficiently they need to switch between several applications on the same or different devices. Indeed, most of today's jobs require people to switch between different media sources, and for some professions multitasking is an inherent feature. A programmer, for instance, writes code while at the same time paying attention to other tasks, such as running another program, responding to email, instant messaging, using multiple display systems etc. [e.g., Sanjram and Khan (2011) and Vasilescu et al. (2016)].

A similar assumption is behind many modern technological devices which promote multitasking and thus claim to increase people's effectiveness. This assumption is readily seen in many advertisements for smartphones, tablets, Wi-Fi-connected laptops, Blackberry phones, and so on: they are intended to increase productivity (i.e., goal accomplishment). Many of the latest versions of various operating systems (like Windows and iOS) even encourage their users to function in a multitasking way, which is supported by the possibility of dividing the screen into several windows and working in several applications simultaneously.

However, people engage in media multitasking not only to satisfy their professional goals but also to—if not primarily—meet their social and emotional goals. Studies show that one of the main reasons people use their mobile devices for is to communicate with others (e.g., the most prevalent reasons for in-class mobile phone use are texting, using social media, or emailing; Burns and Lohenry, 2010; Junco, 2012; Junco and Cotten, 2012). Brooks (2015) demonstrated that the need for connection with family and friends via social media consumes 28% of a worker's day, which further leads to inefficiency in task performance and causes a problem with mentally returning to the main task (Brooks, 2015). Also, Szumowska and Kruglanski (under review) demonstrated that one of the most frequent reasons students use smartphones in class is to communicate with others.

Also, media multitasking serves important emotional goals. Research shows that people engage in additional media tasks in order to regulate their affect and obtain emotional gratification. For instance, multitasking behaviors are positively correlated with enjoyment (Chinchanachokchai et al., 2015; Xu and David, 2018) and sensation seeking (Jeong and Fishbein, 2007; Sanbonmatsu et al., 2013). On the other hand, research has shown that boredom (Wang et al., 2006), fatigue or anxiety induced by arduous activities all trigger multitasking (e.g., Lin, 2013). Further, the results of the study by Judd and Kennedy (2011) suggest that media multitasking might be used to cope with uncertainty. The authors showed that male and international students who do not study in their country of origin are particularly inclined to engage in frequent media multitasking (compared to their female and local counterparts). According to their explanation, this is because starting a course of study in another country increases the feeling of uncertainty, which is in turn associated with a higher level of multitasking. At the same time, students who engage in multitasking most often are those who entered university immediately after high school. Thus, people might engage in media multitasking to seek positive and avoid negative emotions.

A special emotion which is often discussed in the context of media multitasking is the fear of missing out (FOMO). FOMO is defined as a pervasive apprehension that others might be having rewarding experiences which one is missing, and it is characterized by the desire to stay continually connected with what others are doing (Przybylski et al., 2013). Importantly, FOMO is closely associated with susceptibility to various types of distractors, primarily checking social media.

Therefore, engaging with social media while performing other tasks at the same time can be a way to cope with one's FOMO. This is all the more important because this tendency to anxiously engage in excessive activities on social media can cause a decline in the quality of functioning at work or school (Judd and Kennedy, 2011; Przybylski et al., 2013). This suggests that media multitasking may be a behavior that is strategically undertaken to cope with one's emotions (such as FOMO), but this comes at the expense of performance decrements in other areas.

This approach is also in line with the postulates of other researchers who claim that digital multitasking is undertaken in the service of a person's goals and needs. Bardhi et al. (2010) identified various benefits of media multitasking which are described by high media multitaskers: (1) control of media consumption; (2) efficiency—when the media content in each medium is related; (3) engagement in multiple media stimuli; and (4) assimilation—easy connection to others. In a literature review, Garaus (2019) described several different groups of needs which are associated with media multitasking. The first group concerns cognitive needs, such as higher efficiency, information seeking, ease of use, stronger feelings of control and convenience. The second group concerns emotional needs such as sensation seeking and perceived enjoyment. Chang (2016) showed that the sensation-seeking tendency determines the intensity of media use, which finally determines media multitasking behavior. Sensation seekers use more novel and varied forms of media content, whereas individuals low on sensation seeking use forms that are more familiar to them.

People also engage in media multitasking habitually. Habit is often indicated as the reason why people watch TV while performing other tasks [multitasking motives for internet-based multitasking are information seeking and enjoyment (Garaus, 2019)]. People who habitually multitask do not report having any specific goals in mind, such as information seeking, social interaction or enjoyment, and they use a given medium as a routine or to pass time. They also do so to avoid boredom (Sanbonmatsu et al., 2013). However, since passing time, avoiding boredom, or pleasure are also goals, even though they are not always consciously present in one's mind, even habitual media multitasking is strategic in our opinion [see also Kruglanski and Szumowska (2020)].

## Discussion and Conclusions

We have proposed that in order to ascertain whether media multitasking is effective or not, one needs to specify what goal or goals are used as a reference point, how performance is defined (whether subjective or objective measures, and what indices exactly are used) and what consequences we analyze (whether we are interested in immediate, short-term or long-term outcomes). Depending on these differentiations, media multitasking can be seen as both a strategic behavior undertaken to accomplish one's goals as well as a self-regulatory failure.

Most of the research on media multitasking focuses primarily on the aspect of efficiency. In laboratory conditions, the level of knowledge acquisition or the performance of cognitive tasks are typically measured. However, some contexts and motivations may be overlooked when focusing only on one aspect. As we have argued in the present paper, it is important to consider the goals of a person who works in the multitasking mode and use these goals to assess the person's efficiency. As argued above, people multitask in order to attain their professional and academic goals (and the tasks that serve these goals), but they also do so to avoid boredom, satisfy the need for social contact, or to feel more diligent. If we interpret the effectiveness of media multitasking only from the point of view of the level of performance of laboratory tasks, we will unfortunately miss some important aspects: for example, from the point of view of a given person it may be more important to establish contact with a friend than to study for an exam or score 100% in a test. Such a person might be less motivated to study rather than engage in communication with a friend, which likely is reflected in their progress on both activities.

The above issue is related to the gap between the effects of media multitasking found in real-life and in laboratory experiments; these have not been discussed here, but they have been broadly discussed elsewhere [e.g., Salvucci and Taatgen (2011) and Wiradhany and Nieuwenstein (2017)]. It is possible that the tasks and goals that people are presented with in the lab are not necessarily similar to those in their everyday lives. And even when people adopt the goals presented to them in the laboratory, the importance they ascribe to these goals might vary. This matters as people adjust the performance strategy to the importance of their goals (Judd and Kennedy, 2011; Szumowska and Kruglanski, under review). In addition, research by Kononova et al. (2016) shows that forcing multitasking leads to ineffectiveness, and it is only when individuals themselves regulate the way in which they act that they multitask effectively. Regulation of one's performance strategy is typically possible in everyday life but is often limited in laboratory settings, which might at least partially explain the gap.

What also determines whether multitasking will be a failure or an effective strategy for completing a task or fulfilling a given need is one's level of self-regulation ability. This ability is crucial when we have to manage several activities at once, which may be manifested in ignoring distractors, changing a strategy of action, or abandoning certain activities that we consider less important at a given moment [e.g., Neal et al. (2017)]. Failure to ignore less important stimuli may result in poor performance. Hence, the level of self-regulation ability is important in managing media multitasking and it may be a crucial factor in determining whether media multitasking will be a failure or a strategic behavior. This hypothesis may be supported by our own research (Szumowska et al., 2018) in which we showed that people with a low level of self-regulation ability were unable to concentrate on the tasks at hand and switched between websites more often (each task was presented in a separate browser tab, thus switches between websites represent switches between tasks). People with a high level of self-regulation ability, on the other hand, were able to keep their attention on the task for longer, which manifested itself in less frequent switching between websites. A similar effect was obtained when the subjects were working in an imposed sequential condition. In a condition in which they could freely switch between tasks, people with a high level of media multitasking performed worse and switched between websites more often than people with a low level of media multitasking. No differences in performance were observed in the sequential condition. This shows that the ability to organize activities, as represented by both personal self-regulation and external regulations, can affect the effectiveness of multitasking. Self-regulation ability can also help identify the state in which we begin to fall into the illusion of productivity or become addicted to social media. People are able to identify when the multitasking mode of functioning is starting to become burdensome for them and therefore change their operating strategy.

## Practical Implications

Electronic devices (computers, tablets, smartphones, etc.) have become an integral part of our lives. Therefore, we need to get used to the fact that media multitasking is “the new normal” (Courage et al., 2015) and we are not likely to change this. So, we have to start thinking about how to weave this activity into everyday functioning and how to benefit from it effectively. Instead of trying to forbid modern devices in the classroom, it is better to promote active learning by using them. Engaging more deeply in learning by using modern technology is more effective. It also important to remember that media multitasking is more productive when there is a relationship between all activities, as suggested by the results of a study by Angell et al. (2016). It shows that the indicators of efficiency (recall and recognition) are higher when the second task, which is performed at the same time as the main activity, is contextually related; for example, watching a movie and checking information about an actor in it. They also showed the same pattern of results when the second task entailed a high level of social accountability (e.g., texting or sending tweets about this movie). Thus, information-processing frames are very important and can lead to more or less effective performance.

We also need to consider that media multitasking behavior is undertaken for various reasons. By understanding the motivations and being mindful of how often and for what purposes we multitask, we can avoid the illusion of productivity or eliminate unwanted digital behaviors such as constantly checking social media notifications. The level of self-regulation can help in this kind of monitoring. Also, external aids in the form of task- and interruption-managing programs and applications can be useful. As May and Elder (2018) mentioned, structured tasks with specific and clearly indicated technology usage requirements are less likely to provoke off-task multitasking. It is also worth mentioning that there is a tendency to overestimate one's multitasking ability (Sanbonmatsu et al., 2013). This is why education and developing self-regulation skills are crucial for coping with the negative outcomes of media multitasking.

## Author Contributions

All authors conceived the presented idea, developed the theory, and contributed to the final manuscript. ES provided the motivational framework.

## Conflict of Interest

The authors declare that the research was conducted in the absence of any commercial or financial relationships that could be construed as a potential conflict of interest.
